# Supplementary figures and images for: Assessment of sediment porewater toxicity in Biscayne National Park with sea urchin (Lytechinus variegatus) embryos
Source: PLoS One. 2022 Dec 6;17(12):e0278695. doi: 10.1371/journal.pone.0278695 (PMC9725154; doi:10.1371/journal.pone.0278695)

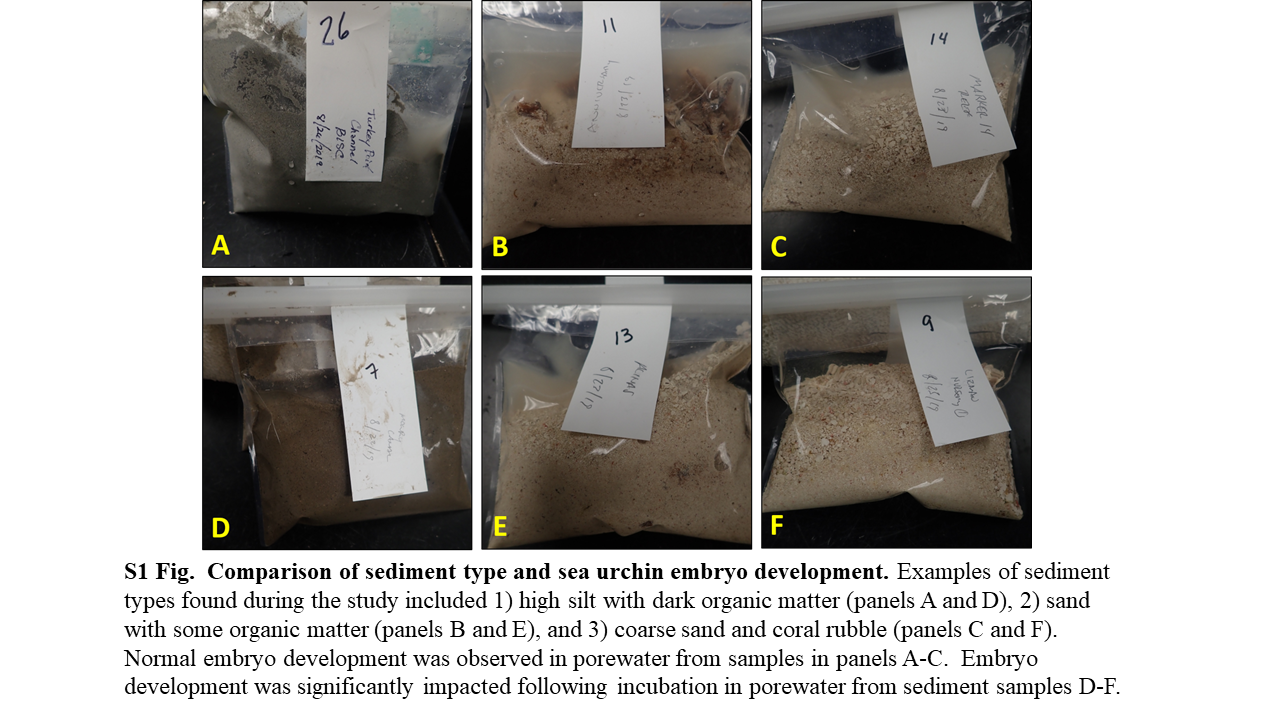

Supplement: S1 Fig — Examples of sediment types found during the study included 1) high silt with dark organic matter (panels A and D), 2) sand with some organic matter (panels B and E), and 3) coarse sand and coral rubble (panels C and F). Normal embryo development was observed in porewater from samples in panels A-C. Embryo development was significantly impacted following incubation in porewater from sediment samples D-F. (TIF) [file pone.0278695.s002.tif]
