# Supplementary material for: Assessment of sediment porewater toxicity in Biscayne National Park with sea urchin (Lytechinus variegatus) embryos
Source: PLoS One. 2022 Dec 6;17(12):e0278695. doi: 10.1371/journal.pone.0278695 (PMC9725154; doi:10.1371/journal.pone.0278695)
Supplement: Attachment — Submitted filename: Response to reviewers.docx [file pone.0278695.s003.docx]

**Response to Reviewers**

Journal Requirements:

When submitting your revision, we need you to address these additional requirements.

1. Please ensure that your manuscript meets PLOS ONE's style requirements, including those for file naming. The PLOS ONE style templates can be found at

<https://journals.plos.org/plosone/s/file?id=wjVg/PLOSOne_formatting_sample_main_body.pdf> and

<https://journals.plos.org/plosone/s/file?id=ba62/PLOSOne_formatting_sample_title_authors_affiliations.pdf>

**Authors’ response: We have corrected formatting for file naming, author affiliations and references (brackets instead of parentheses).**

2. Please include your full ethics statement in the ‘Methods’ section of your manuscript file. In your statement, please include the full name of the IRB or ethics committee who approved or waived your study, as well as whether or not you obtained informed written or verbal consent. If consent was waived for your study, please include this information in your statement as well.

**Authors’ response: We have added an ethics statement to the ‘Materials and methods’ section to comply with this requirement.**

3. We note that Figure 3 in your submission contain satellite images which may be copyrighted. All PLOS content is published under the Creative Commons Attribution License (CC BY 4.0), which means that the manuscript, images, and Supporting Information files will be freely available online, and any third party is permitted to access, download, copy, distribute, and use these materials in any way, even commercially, with proper attribution. For these reasons, we cannot publish previously copyrighted maps or satellite images created using proprietary data, such as Google software (Google Maps, Street View, and Earth). For more information, see our copyright guidelines: <http://journals.plos.org/plosone/s/licenses-and-copyright>.

 We require you to either (1) present written permission from the copyright holder to publish these figures specifically under the CC BY 4.0 license, or (2) remove the figures from your submission:

a. You may seek permission from the original copyright holder of Figure 3 to publish the content specifically under the CC BY 4.0 license.

We recommend that you contact the original copyright holder with the Content Permission Form (<http://journals.plos.org/plosone/s/file?id=7c09/content-permission-form.pdf>) and the following text:

“I request permission for the open-access journal PLOS ONE to publish XXX under the Creative Commons Attribution License (CCAL) CC BY 4.0 (<http://creativecommons.org/licenses/by/4.0/>). Please be aware that this license allows unrestricted use and distribution, even commercially, by third parties. Please reply and provide explicit written permission to publish XXX under a CC BY license and complete the attached form.”

Please upload the completed Content Permission Form or other proof of granted permissions as an "Other" file with your submission.

In the figure caption of the copyrighted figure, please include the following text: “Reprinted from [ref] under a CC BY license, with permission from [name of publisher], original copyright [original copyright year].”

 b. If you are unable to obtain permission from the original copyright holder to publish these figures under the CC BY 4.0 license or if the copyright holder’s requirements are incompatible with the CC BY 4.0 license, please either i) remove the figure or ii) supply a replacement figure that complies with the CC BY 4.0 license. Please check copyright information on all replacement figures and update the figure caption with source information. If applicable, please specify in the figure caption text when a figure is similar but not identical to the original image and is therefore for illustrative purposes only.

The following resources for replacing copyrighted map figures may be helpful:

USGS National Map Viewer (public domain): <http://viewer.nationalmap.gov/viewer/>

The Gateway to Astronaut Photography of Earth (public domain): <http://eol.jsc.nasa.gov/sseop/clickmap/>

Maps at the CIA (public domain): <https://www.cia.gov/library/publications/the-world-factbook/index.html> and <https://www.cia.gov/library/publications/cia-maps-publications/index.html>

NASA Earth Observatory (public domain): <http://earthobservatory.nasa.gov/>

Landsat: <http://landsat.visibleearth.nasa.gov/>

USGS EROS (Earth Resources Observatory and Science (EROS) Center) (public domain): [http://eros.usgs.gov/#](http://eros.usgs.gov/)

Natural Earth (public domain): <http://www.naturalearthdata.com/>

**Authors’ response: We have changed the Fig 3 map of Biscayne National Park to one that is in the public domain (source, National Park Service).**


[Note: HTML markup is below. Please do not edit.]

Reviewers' comments:

Reviewer's Responses to Questions

**Comments to the Author**

1. Is the manuscript technically sound, and do the data support the conclusions?

The manuscript must describe a technically sound piece of scientific research with data that supports the conclusions. Experiments must have been conducted rigorously, with appropriate controls, replication, and sample sizes. The conclusions must be drawn appropriately based on the data presented.

Reviewer #1: Yes

Reviewer #2: Partly

2. Has the statistical analysis been performed appropriately and rigorously?

Reviewer #1: Yes

Reviewer #2: Yes

3. Have the authors made all data underlying the findings in their manuscript fully available?

The [PLOS Data policy](http://www.plosone.org/static/policies.action#sharing) requires authors to make all data underlying the findings described in their manuscript fully available without restriction, with rare exception (please refer to the Data Availability Statement in the manuscript PDF file). The data should be provided as part of the manuscript or its supporting information, or deposited to a public repository. For example, in addition to summary statistics, the data points behind means, medians and variance measures should be available. If there are restrictions on publicly sharing data—e.g. participant privacy or use of data from a third party—those must be specified.

Reviewer #1: Yes

Reviewer #2: Yes

4. Is the manuscript presented in an intelligible fashion and written in standard English?

PLOS ONE does not copyedit accepted manuscripts, so the language in submitted articles must be clear, correct, and unambiguous. Any typographical or grammatical errors should be corrected at revision, so please note any specific errors here.

Reviewer #1: Yes

Reviewer #2: Yes

5. Review Comments to the Author

Please use the space provided to explain your answers to the questions above. You may also include additional comments for the author, including concerns about dual publication, research ethics, or publication ethics. (Please upload your review as an attachment if it exceeds 20,000 characters)

Reviewer #1: 1. Generally, the ionic composition and redox condition of porewater are different from normal seawater. Whether sediment porewater itself is suitable for sea urchin embryo development when salinity and ammonia were not considered ?

**Authors’ response: The authors would like to thank Reviewer 1 for the critical comments. The sea urchin embryo development test is a standard method (ASTM and US EPA) and has been used by other research groups to assess the toxicity of marine porewaters. Biscayne National Park is a habitat for *Lytechinus variegatus*, so this is a logical species to select for this toxicity assay. Normal sea urchin embryo development was reported for three sites in this study and across sediment types (see #2 below), indicating that the ionic and redox composition of porewater from various sediment types is suitable for urchin embryo development. Both salinity and ammonia were measured in all porewater samples prior to the test. Salinity was adjusted to within 1 ppt of the artificial seawater control, a range which does not negatively affect *L. variegatus* embryo development. Total ammonia nitrogen was measured in each sample, however it is not easily removed without potentially altering the chemical characteristics of the porewater (and altering any potential chemical contaminants). We therefore decided to report ammonia and state the caveat that ammonia could contribute to the toxicity at a site where it was above the reported EC_50_ or US EPA chronic criterion. References are given for both criteria. We have additionally performed a Spearman ranked correlation test of sediment porewater ammonia concentration and percent normal embryo development, per comments below. We found no correlation between these parameters and have reported these results in the revised manuscript.**

2. Please demonstrate the type of sediment if any. Whether sea urchin embryo development in porewater is related to sediment type?

**Authors’ response: Sediment type varied across the sample sets, from a relatively high silt and organic matter content (noted in images and visual inspection) to more coarse coral rubble, typically found in and around stony coral reef areas. We observed normal sea urchin embryo development across the range of sediment types, indicating that this parameter had no impact on the assay. We have included a supplemental figure with example images from six sites, two of which were high in silt content and darker in color (possible higher organic matter content), two which were sand and silt with some organic matter and two which were of the consistency of coarse sand, shell and coral rubble. Each sediment type in the figure had both normal embryo development (panels A -C) and impacted embryo development (panels D-F).**

3. Correlation analysis between embryo development and ammonia is needed.

**Authors’ response: A Spearman’s rank correlation (data were not normally distributed) was computed to assess the relationship between ammonia concentration in the sediment porewater and sea urchin percent normal embryo development. There was a negative correlation between the two variables, r(23) = -.27, p = 0.20. We have reported this in the Results and discussion section of the revised manuscript.**

4. Please state the function of SDS Controls in the section of Materials and methods.

**Authors’ response: We stated that the sodium dodecyl sulfate (SDS) functions as the reference toxicant (assay positive control, Materials and methods, lines 168-169 of the original manuscript).**

5. Toxicity screening is needed for further works.

**Authors’ response: The authors agree. Future work will incorporate a toxicity identification evaluation (TIE) to narrow the class of target analytes, followed by targeted analytical chemistry. This will help inform Park managers of potential risks to Park resources and guide management actions to reduce those risks.**

Reviewer #2: Generally, the article is easy to follow. The authors collected sediment samples and performed the SUETOX test on the extracted porewater. The article discusses the toxicity of sediments and points out the need to identify the source. This is a general investigation and the following questions need to be carefully addressed.

1. The title is not specific enough. The target biota should at least be involved.

**Authors’ response: The authors would like to thank Reviewer 2 for all critical comments. We have revised the title to be more specific and have included the target test organism.**

2. Why didn’t the authors analyze the contaminants in the target sediments? It would be much reasonable to at least show which contaminants were mainly in the target sediments.

**Authors’ response: This study was undertaken to avoid costs associated with unnecessary and/or non-targeted analytical chemistry analyses. As mentioned above (Reviewer 1, comment #5), future work will entail Level 1 screening using a toxicity identification evaluation (TIE) and follow up chemistry analyses to determine the nature of the toxicity.**

L114 Do authors have any data to support the expectation?

**Authors’ response: Site selection was performed by National Park Service personnel and based upon visual observations for those areas of high recreational use. Marinas and boat harbors were selected to see if those sites exhibited toxicity related to boat use. Coral reef and coral nursery habitats were selected since those are a protected resource within the Park jurisdiction, and the Park is tasked with protecting those resources.**

L117 What’s the full name of NPS?

**Authors’ response: We have changed this abbreviation to ‘National Park Service’**

L122 The “x” should be a multiplication sign.

**Authors’ response: We have changed the ‘x’ to ‘by’ since this is the dimension of the storage vessel.**

L123, 125 The hyphen should be a minus.

**Authors’ response: We inserted a minus symbol, however in this font, this symbol has a dual role as a hyphen.**

L143 No need to set all the parameters for Mowry Canal to bold.

**Authors’ response: The authors agree and have eliminated bold text for all parameters except the ammonia (UAN) concentration.**

L153 Should be “fed with”?

**Authors’ response: We have added ‘with’ to the sentence indicated.**

L232 The red labels were vague.

**Authors’ response: The authors agree. We have revised Fig 3 and have changed the font color to black for all site names to address this issue.**

6. PLOS authors have the option to publish the peer review history of their article ([what does this mean?](https://journals.plos.org/plosone/s/editorial-and-peer-review-process#loc-peer-review-history)). If published, this will include your full peer review and any attached files.

If you choose “no”, your identity will remain anonymous but your review may still be made public.

**Do you want your identity to be public for this peer review?** For information about this choice, including consent withdrawal, please see our [Privacy Policy](https://www.plos.org/privacy-policy).

Reviewer #1: No

Reviewer #2: **Yes:** Ciara Chun Chen
